# The role of peptide and DNA vaccines in myeloid leukemia immunotherapy

**DOI:** 10.1186/1475-2867-13-13

**Published:** 2013-02-11

**Authors:** Chen Lin, Yangqiu Li

**Affiliations:** 1 Department of Microbiology and Immunology, Medical College, Jinan University, Guangzhou, 510632, China; 2 Institute of Hematology, Medical College, Jinan University, Guangzhou, 510632, China; 3 Key Laboratory for Regenerative Medicine of Ministry of Education, Jinan University, Guangzhou, 510632, China

**Keywords:** DNA vaccine, Peptide vaccine, Leukemia-associated antigen, Myeloid leukemia, Immunotherapy

## Abstract

While chemotherapy and targeted therapy are successful in inducing the remission of myeloid leukemia as acute myeloid leukemia (AML) and chronic myeloid leukemia (CML), the disease remains largely incurable. This observation is likely due to the drug resistance of leukemic cells, which are responsible for disease relapse. Myeloid leukemia vaccines may most likely be beneficial for eradicating minimal residual disease after treatment with chemotherapy or targeted therapy. Several targeted immunotherapies using leukemia vaccines have been heavily investigated in clinical and preclinical trials. This review will focus on peptides and DNA vaccines in the context of myeloid leukemias, and optimal strategies for enhancing the efficacy of vaccines based on myeloid leukemia immunization are also summarized.

## Introduction

While chemotherapy and targeted therapy are successful at inducing myeloid leukemia remission, the disease remains largely incurable. We have come to realize that immunotherapy may result in a cure for the disease [1-5]. The goal of immunotherapy in myeloid leukemia is to boost the patient immune system or confer immunity with T cells, dendritic cells (DC), NK cells or monoclonal antibodies.

Myeloid leukemia vaccines are most likely beneficial for eradicating minimal residual disease after chemotherapy or targeted therapy [6] although the suppressed immune status of patients who receive these treatments may influence their vaccine response. However, targeted immunotherapy using leukemia vaccines has been heavily investigated because these vaccines elicit specific immune responses against leukemia cells while sparing normal tissue. Optimal immunotherapy target antigens are leukemia-specific antigens that are exclusively expressed by leukemia cells, are absent in normal tissues, and can elicit potent immune responses; however, with the exception of the BCR-ABL and PML-RARα fusion proteins, such leukemia-specific antigens are rare in other myeloid leukemias. Leukemia-associated antigens (LAAs), such as Wilms’ tumor 1 (WT1) antigen, proteinase-3 peptide, preferentially expressed antigen of melanoma (PRAME), and receptor for hyaluronic acid-mediated motility (RHAMM), are preferentially expressed by leukemia cells but are also expressed by normal tissues, albeit to a lesser degree [7].

Peptide vaccines have been developed against tumor-specific and leukemia-associated self-antigens. Numerous clinical trials involving peptide vaccines have been performed with limited success [8-11]. It has become clear that exogenous peptides alone fail to activate effective CD8+ T cell levels, and if induced, they tend to be transient in patients with a weakened or tolerized immune system. Therefore, DNA vaccines present an attractive, alternative strategy for peptide vaccination [12-15].

DNA vaccines are bacterial plasmids constructed to express an encoded protein following in vivo administration and subsequent cell transfection. DNA vaccines have many advantages for tumor antigens. First, to some extent, encoded antigens can enter the processing and presentation pathways of the immune system and induce adaptive (antibodies, helper T cells, and cytotoxic lymphocytes (CTLs) ) and innate immune responses in a manner similar to natural infection. Second, non-specific innate immunity stimulation, which can act against tumor growth, is provided by the bacterial DNA backbone. Activation of the innate immune system is the first step in the induction of immunity against weak tumor antigens. In fact, the presence of CD4+ T cells is also a critical requirement for the generation of effector CTL responses [16-21].

Results from animal models and initial clinical trials are promising, but none have been translated into successful standardized clinical treatments thus far, emphasizing the many differences between animal models and patients [22-25]. This article will summarize peptide and DNA vaccines in the context of myeloid leukemias.

## Leukemia-specific antigens and LAA-derived vaccines in myeloid leukemias

During the past decade, the understanding of the immunology of myeloid leukemia has raised hopes that this disease may be curable with immunotherapeutic approaches. The optimal target antigens for immunotherapy are leukemia-specific antigens. It is well known that abnormal fusion proteins resulting from chromosomal translocations can serve as relative leukemia-specific CTL response targets including the BCR-ABL and PML-RARα fusion proteins. Other attractive LAAs, including WT1 antigen, proteinase-3 peptide, PRAME, and RHAMM, which are self-antigens overexpressed in leukemia cells, can also serve as leukemia-associated targets for immune responses [5,7].

### BCR-ABL vaccines for BCR-ABL + leukemia

BCR-ABL fusion protein expression is unique to CML leukemic cells and a portion of acute lymphocytic leukemia (ALL) cells and derives from a t(9;22) translocation. Although imatinib is the standard treatment for CML, not all patients reach complete cytogenetic remission (CCR) and most maintain detectable disease at the molecular level. Therefore, targeting the BCR-ABL-derived p210 fusion protein may be an active and specific immunotherapy for improving outcome. Although immune dysfunction has been demonstrated in many patients with CML [26], it has been recently reported that immunological responses to the e14a2 BCR-ABL peptide were statistically similar to that of 5 healthy subjects with the same vaccination schedule as patients with CML [27].

A number of clinical trials have been performed with vaccines based on the p210 BCR-ABL fusion protein. A tumor-specific BCR-ABL breakpoint peptide-derived vaccine may be safely administered and reliably elicit measurable peptide-specific immune responses in patients, including those that received a bone marrow transplant, interferon, or imatinib [28-31].

In particular, BCR-ABL peptide vaccination not only stimulated specific tumor-specific immune responses in patients with CML, it also allowed a reduction in the imatinib dose given and the benefit persistence of a CML complete molecular response [32].

Breccia et al. reported the effects of p210 vaccination (CMLVAX100) in two patients with residual molecular disease after high dose imatinib (600 or 800 mg). CMLVAX100 comprised 100 μg each for five p210b3a2 breakpoint-derived peptides including four HLA class I binding peptides (ATGFKQSSK, A11-binding; KQSSKALQR, A3-binding; HSATGFKQSSK, A3 and A11-binding; and GFKQ-SSKAL, B8-binding) and a 25 amino acid peptide IVHSAT-GFKQSSKALQRP VASDFEP (b3a2-25) with binding motifs for HLA class II DR1, DR4, and DR11. In case #1, vaccination was begun in a 42-year-old male with chronic phase (CP) CML with intermediate Sokal risk (b2a2 transcript type) 54 months after beginning imatinib treatment with CML VAXb2a2-25, a p210 b2a2 peptide (sequence: TVHSIPLTINKEEALQRPVASDFEP), at a 100 μg dose, while in case #2, a 44-year-old male with high Sokal risk CP CML (b3a2 transcript type) had vaccination treatment started 20 months after high dose imatinib therapy. The use of a transcript-related vaccine enabled a reduction in the imatinib dose and the concomitant realization of a persistent molecular response [33].

In addition to the BCR-ABL peptide, a series of DNA vaccines based on the BCR-ABL fusion gene were developed and tested in mice or in vitro. For example, plasmids encoding the complete BCR-ABL fusion gene were capable of inducing protection against challenges from B210 or 12B1 cells. No BCR-ABL-positive cells were found in the liver, spleen or bone marrow of successfully immunized animals [34].

### PML-RARα vaccines for acute promyelocytic leukemia

Acute promyelocytic leukemia (APL) is associated with a t(15;17) (q22;q11.2) chromosomal translocation and involves the retinoic acid receptor α (RARα) gene on chromosome 17 and its fusion partners including the promyelocytic leukemia (PML) gene on chromosome 15. The fusion gene encodes the corresponding fusion protein thought to play a fundamental role in leukemogenesis. The PML-RARα fusion protein is presented at the cell surface by MHC molecules and may induce a tumor-specific T-cell response in patients [35]. Therefore, the PML-RARα fusion protein or fusion gene may be selected as a specific leukemia antigen for inducing a specific immune response in patients with APL to further prolong the remission duration and eradicate minimal residual disease.

Although there were few preclinical or clinical trials involving vaccination with a PML-RARα peptide against leukemic cells, DNA vaccines specifically targeting the PML-RARα fusion protein can have a pronounced effect on survival alone and in combination with all-trans retinoic acid (ATRA) in animal models [36,37]. Immunocompetent mice given DNA vaccines encoding various portions of the BCR-1 PML-RARα fusion protein could clear engrafted APL cells. These data demonstrate that the tumor-specific immune clearance of APL cells occurs in mice [38]. Another study indicated that a DNA vaccine containing the PML-RARα segment and tetanus fragment C (FrC) sequences could increase survival times when used alone or in combination with all-trans retinoic acid (ATRA) in a mouse APL model [37], which is similar to a study that demonstrated that DNA vaccination with ATRA confers the effective boosting of interferon-gamma-producing and cytotoxic T-cells in leukemic mice [39].

### WT1 vaccines for myeloid leukemias

WT1 is an oncogenic protein expressed by the Wilms’ tumor gene that is overexpressed in the majority of AMLs and CMLs. WT1 expression in progenitor cells is minimal or absent, and the limited WT1 tissue expression in adults suggests that WT1 may be a therapeutic leukemia target.

Results from an immune response WT1 vaccination study provides immunologic, molecular, and preliminary evidence for its potential clinical efficacy in AML patients [40]. Ochsenreither et al. identified WT1-specific T-cell clones from a patient with recurrent AML vaccinated with a WT1 peptide (epitope: 126–134) who achieved a complete remission (CR) lasting for 12 months. In addition, a specific predominant T-cell clone was identified during clinical remission, and it was increased in the PB and BM after 8 vaccinations [41]. This predominant TCR Vβ11 clone could be found in other AML patients vaccinated with the same WT1 peptide. Thus, WT1-specific T-cell populations with proven clinical impact in WT1-vaccinated patients may be considered as an immune response biomarker after vaccination [42].

Increasing data demonstrate that WT1 peptide vaccines may become a safe and cure-oriented therapy for patients with CML who have residual disease. WT1 peptide vaccines may also be used for patients with CML who have residual disease, particularly those who are resistant to BCR-ABL tyrosine kinase inhibitors such as imatinib. In an imatinib-treated patient with CML who was administered a WT1 peptide vaccine, a decrease in BCR-ABL mRNA levels was associated with an increased frequency of WT1-specific CTLs in the patient’s peripheral blood [43]. The WT1 peptide was administered 22 times over 18 months in a clinical trial for a CML patient who was being treated with imatinib, and the BCR-ABL transcripts remarkably decreased to a major molecular response level after WT1 peptide administration, indicating its beneficial effects on minimal residual disease [44].

WT1 as an immunotherapeutic target of a designed DNA fusion vaccine was also evaluated. Plasmid DNA encoding the full-length murine WT1 gene was intramuscularly injected into C57BL/6 mice. Mice vaccinated with WT1 plasmid DNA elicited CTLs against the WT1 protein, and the CTLs specifically killed WT1-expressing tumor cells in a MHC class I-restricted manner [45]. Another preclinical experiment demonstrated that three p.DOM-peptide vaccines, each encoding a different WT1-derived, HLA-A2-restricted epitope, induced CTLs in humanized transgenic mice expressing chimeric HLA-A2 without affecting hematopoietic stem cells, and these CTLs killed human leukemia cells in vitro. These results indicate that rationally designed DNA vaccines encoding WT1-derived epitopes, particularly WT1 (37–45), have the potential to induce/expand functional tumor-specific cytotoxic responses in patients with cancer [46].

### Potential LAA vaccines

#### PRAME

PRAME is overexpressed in many hematologic malignancies, including AML [47] and CML [9], but is absent in normal tissues, including hematopoietic progenitor cells, and may be an appropriate candidate for T cell-mediated immunotherapy. PRAME CTLs from healthy donors and patients with CML were induced by HLA-A*02-restricted PRAME-peptides under optimized culture conditions. These CTLs released IFNγ in response to the PRAME peptides and lysed PRAME peptide-loaded cells in an MHC-restricted fashion [9]. Moreover, using professional and artificial antigen-presenting cells loaded with a peptide library spanning the PRAME protein that consisted of 125 synthetic pentadecapeptides overlapping by 11 amino acids, polyclonal, PRAME-specific CTL lines were successfully generated and elicited high-avidity CTLs with a high proportion of the cells recognizing a previously uninvestigated HLA-A*02-restricted epitope, P435-9mer (NLTHVLYPV). The cytotoxic activity of PRAME-specific CTLs was directed not only against leukemic blasts but also against leukemic progenitor cells, which have been implicated in leukemia relapse. These PRAME-directed CTLs did not affect normal hematopoietic progenitors, indicating that this approach may be of value for PRAME + hematologic malignancy immunotherapy [48].

PRAME peptides were first used in combination with the WT1 peptide vaccine in the clinic, and vaccinations could also improve the targeting of differentiated progeny and suboptimally benefit patients responding to tyrosine kinase inhibitors or enhance GVL effects in patients with SCT [1].

#### PR1

PR1 is a 9–amino acid HLA-A*0201–restricted peptide derived from proteinase 3, which is present at a high concentration in the primary granules of acute and chronic myeloid leukemia blasts. PR1 induced myeloid leukemia–specific CTL responses that selectively killed leukemic CD34^+^ cells in vitro [49], suggesting that it should be relatively easy to boost these immune responses with vaccination. Highly encouraging preliminary data from a phase 1/2 study evaluating PR1 vaccination in patients with myeloid leukemias were presented at the annual meeting of the American Society of Hematology in 2004 [50].

In clinical trials, PR1 was frequently used with the WT1 vaccine as a combination immunization strategy in myeloid leukemia patients. For example, in a small trial, 8 patients received one subcutaneous dose each of the PR1 and WT1 vaccines. CD8+ T cells directed against PR1 or WT1 were detected in all patients after a single vaccination. After vaccination, the emergence of PR1- or specific CD8+ T cells was associated with a decrease in WT1 mRNA expression as a marker for minimal residual disease. This result indicated that a combined PR1 and WT1 vaccine is immunogenic and safe [51].

#### RHAMM

RHAMM was identified as one of the most promising LAAs in AML. Recently, limited clinical data demonstrated that high-dose RHAMM-R3 peptide vaccination in patients with AML could induce positive immunological responses [52]. Moreover, RHAMM not only represents a promising LAA with specific T-cell responses in AML, it is also a probable prognostic factor if it is assessed on blasts in situ [53]. These results support the further study of immunization strategies using RHAMM alone or combination with other LAA vaccines in leukemia patients.

#### HSP

Heat shock proteins (HSPs) are highly conserved molecules with many immunological functions. These proteins are highly immunogenic and play an important role in cancer immunotherapy. It was demonstrated that HSP70PC vaccination is feasible and safe. When combined with imatinib mesylate, it is associated with immunologic and potential clinical responses against CML-CP. In a clinical trial, Hsp70PCs were purified from leukopheresed peripheral blood mononuclear cells and administered via eight weekly intradermal injections at 50μg /dose without adjuvant. A significant correlation between the clinical and immunologic responses was observed. Cytogenetic responses were observed in the bone marrow BCR-ABL + leukemia cells in 13 of 20 patients, despite imatinib treatment for all except one patient. In addition, the frequency of CML-specific, IFNγ-producing and IFNγ-secreting NK cells in blood from 9 of 16 patients increased [54].

### Enhancing immunogenicity

The ability of leukemia vaccines to elicit an immune response to cancers has been well documented in clinical trials and numerous animal models. However, effective anti-leukemia immune responses are hampered by the weak immunogenicity of leukemic cells. Most ongoing clinical trials have confirmed that the immune response to the majority of clinically tested DNA vaccines is poor, and further optimization using adjuvant delivery systems or prime-boost-based schedules is a major challenge. Much effort has been made to identify agents that could increase the immunogenicity of leukemic cells and activate the immune system. Genetic adjuvants are usually cytokine genes, which provide general immune stimulation and may also bias the immune response toward a Th1 or Th2 type.

#### GM-CSF

Granulocyte-macrophage colony stimulating factor (GM-CSF) is considered to be the most effective immunostimulating factor [55]. For example, a GM-CSF vaccination was combined with the WT1.126-134 peptide for patients with AML. Seventeen patients with AML and 2 patients with refractory anemia with excess blasts received a median of 11 vaccinations, and treatment was well tolerated. The objective responses in the patients with AML have been detected. WT1 mRNA levels decreased at least 3-fold from baseline in 35% of the patients. WT1-specific T cells increased in the blood or bone marrow in a portion of the patients with a median bone marrow frequency of 0.18% at baseline and 0.41% at week 18 [56].

#### IL-2

Interleukin-2 (IL-2) is one of the immunostimulatory adjuvants used to improve the immune response to vaccine antigens by triggering the activation and proliferation of T cells. A DNA vaccine encoding the PML-RARα fusion gene and the IL-2 gene demonstrated enhance immune responses in PML-RARα-hIL-2-pIRES immunized mice [57].

#### IL-7

IL-7 may influence the growth and differentiation of T-cells, promote T-cell migration into tumor tissue and up-regulate specific cellular immune responses. Vaccines from a plasmid constructed with an mIL-7-BCR-ABL fusion gene induced a better immune response in BALB/c mice immunized with pV BCR-ABL /mIL-7 [58].

#### IL-12

IL12 is an essential cytokine for the generation of a Th1 response, NK cells and CTL stimulation. Cellular immunity could be enhanced by the pBudCE4.1-BCR-ABL-GPI-mIL12 recombinant plasmid. Vaccination by this plasmid resulted in high levels of splenocyte proliferative responses, significant levels of IL-2 and IFNγ, as well as strong CTL responses in vitro. In a murine transplant model, vaccinated mice demonstrated decreased leukemia cell infiltration and reduced BCR-ABL transcript and protein expression in bone marrow cells with a BCR-ABL-GPI-mIL12 vaccine [59].

#### CpG

CpG-ODN is oligodeoxynucleotide containing one or more unmethylated CpG dinucleotides. CpG-ODN could directly and indirectly affect immune cells including DCs, T-cells, B-cells, and NK cells. DNA vaccination against the BCR-ABL oncoprotein with synthetic CpG-ODN and levamisole (LMS) was tested in a mouse tumor model. The overall antitumor effect was enhanced in the tumor model. A high dose of CpG-ODN exhibited a superior adjuvant effect in comparison with all combinations of CpG-ODN and LMS [60].

#### Ubiquitin

Ubiquitin protein fusion results in its targeting to the proteasome and processing through the MHC class I pathway. Plasmids encoding an epitope from the WT1 gene (WT1-126) N-terminally fused to ubiquitin were used to immunize a mouse model. Lymphoproliferative responses following stimulation with the WT1-126 vaccine were observed in all of the immunized mice. Moreover, in vivo cytotoxicity assay results revealed the specific lysis of target cells. Tumor growth decreased, Th1 type cytokines were detected and WT1-126-specific, IFNγ-producing lymphocytes developed in all immunized groups [61].

In addition to the above-described vaccines, exosome-based vaccines were recently reported to potentially be a promising means of prolonging disease-free survival in patients with APL after induction therapy [62]. Exosomes are a family of bioactive vesicles that play important roles in antigen presentation.

## Perspective

An important approach for prolonging remission duration and eradicating minimal residual disease in leukemia is immunotherapy. In myeloid leukemia, preclinical and clinical trials involving vaccination with peptides derived from a number of leukemia antigens, including WT1, BCR-ABL, PML-RARα, PR1 or RHAMM, have demonstrated evidence for immunogenicity, but there are limited data concerning the clinical efficacy of vaccine-based approaches. Most of the DNA vaccines for leukemia are currently in the preclinical stage; thus no DNA cancer vaccine has been licensed for use in humans [63].

Peptide and DNA vaccines derived from myeloid leukemia-specific or leukemia-associated antigens are an attractive approach for immunotherapy to improve overall survival. http://However, optimal strategies for enhancing the efficacy of vaccines based on myeloid leukemia immunization need to be improved. An efficacious vaccine against cancers, including leukemias, will focus on multiple strategies for breaking self-tolerance, such as the improvement of delivery [64,65], codon modification of the antigen [46,66] and costimulation [36,37,57]. Moreover, activation of the powerful immune system to continually attack emerging tumor cells is the goal. Therapeutic efficacy for advanced leukemia treatment surely requires a more potent immune response than prophylaxis, although the presence of large quantities of antigens provides a means for stimulation that may be lacking in the prophylactic setting. Therapeutically vaccinating patients with leukemia, often in the setting of immune damage or tolerance, requires a level of ingenuity beyond that needed for prophylactic vaccines.

## Competing interests

The authors declare that they have no competing interests.

## Authors’ contributions

CL and YQL devised the concept and intellectually contributed to this paper. Both read and approved the final manuscript.
